# Efficacy and safety of asenapine in Asian patients with an acute exacerbation of schizophrenia: a multicentre, randomized, double-blind, 6-week, placebo-controlled study

**DOI:** 10.1007/s00213-016-4295-9

**Published:** 2016-06-08

**Authors:** Toshihiko Kinoshita, Ya-Mei Bai, Jong-Hoon Kim, Mutsuo Miyake, Nobuyuki Oshima

**Affiliations:** Department of Neuropsychiatry, Kansai Medical University, 10-15 Fumizono-cho, Moriguchi, Osaka Japan; Department of Psychiatry, Taipei Veterans General Hospital (TPVGH), Taipei, Taiwan; Department of Psychiatry, College of Medicine, National Yang-Ming University, Taipei, Taiwan; Department of Psychiatry, Gachon University Gil Medical Center, Gachon University School of Medicine, Incheon, Republic of Korea; MSD K.K., Tokyo, Japan

**Keywords:** Schizophrenia, Asenapine, Efficacy, Tolerability

## Abstract

**Rationale:**

Asenapine is a second generation anti-psychotic approved in the USA in 2009 for the treatment of schizophrenia, but its efficacy has not been proven in Asian patients.

**Objectives:**

The objectives of this study are to evaluate the efficacy and tolerability of asenapine in Asian patients experiencing an acute exacerbation of schizophrenia.

**Methods:**

In this prospective, double-blind study, patients in Japan, Korea, and Taiwan were randomized (1:1:1) to asenapine 5 mg twice daily (bid), 10 mg bid or placebo for 6 weeks after a 3- to 7-day washout/screening period. The primary endpoint was the mean change in the positive and negative syndrome scale (PANSS) total score from baseline to day 42/treatment end.

**Results:**

Of the 532 participants randomized, 530 received treatment. The primary endpoint was significantly greater with asenapine 5 and 10 mg bid than with placebo (−12.24 and −14.17 vs. −0.95; *p* < 0.0001). The results of secondary endpoints including PANSS negative subscale scores and PANSS responders at the end of treatment supported the results of the primary endpoint. There were no significant differences in the incidence of treatment-emergent adverse events reported with asenapine 5 and 10 mg bid and placebo (84.6, 80.7, and 81.6 %). There was a mean (± standard deviation) change in weight of −1.76 ± 2.45 kg for placebo, +0.42 ± 2.65 kg for asenapine 5 mg bid, and +0.81 ± 2.89 kg for asenapine 10 mg bid group.

**Conclusions:**

Asenapine was effective and generally well tolerated when used for the treatment of acute exacerbations of schizophrenia in Asian patients.

## Introduction

Schizophrenia is a complex psychiatric disorder associated with variable degrees of functional impairment and social disability (Lublin et al. 2005; Tandon et al. 2009). It is a chronic relapsing disorder, with recurrent exacerbations of positive symptoms against a background of persistent negative symptoms, cognitive dysfunction, and depressive symptoms (Lublin et al. 2005; Tandon et al. 2009).

Anti-psychotic medication is the mainstay of treatment for schizophrenia (van Os and Kapur 2009). Historically, pharmacological treatments have focused on dopamine D_2_ receptor blockade to control the positive symptoms of schizophrenia, while negative and cognitive symptoms often persist (Abi-Dargham 2014; van Os and Kapur 2009). Second generation anti-psychotics, which also inhibit a range of other receptors, have been developed, but they vary in their effectiveness across mood domains (Lublin et al. 2005; Pompili et al. 2011). In addition, second generation anti-psychotics have differing levels of risk for weight gain and hyperprolactinemia (De Hert et al. 2012; Kane 2011; Tandon et al. 2010), which can negatively affect treatment adherence (Kane 2011; Lieberman et al. 2005).

Asenapine is a second generation anti-psychotic with a unique receptor-binding profile that has been approved in the USA for the treatment of schizophrenia (Citrome 2014a; Shahid et al. 2009). It has antagonistic activity at dopaminergic, serotonergic, α-adrenergic, and histaminic receptors but no appreciable affinity for muscarinic receptors (Shahid et al. 2009). The efficacy and safety of asenapine in schizophrenia has been established in a global clinical trial program (Citrome 2014b). In two 6-week fixed-dose trials, asenapine 5 mg twice daily (bid) was more effective than placebo in improving positive and negative syndrome scale (PANSS) total scores (Kay et al. 1987) in patients with acute exacerbations of schizophrenia, with only modest effects on weight and metabolic variables (Kane et al. 2010; Potkin et al. 2007).

In a dedicated study, the pharmacokinetic and safety profiles of asenapine were similar in healthy Japanese and Caucasian subjects (Merck Sharp & Dohme B.V. 2014), meaning no dosage adjustment is required on the basis of race. However, generally Asian subjects account for only a small percentage of the total populations enrolled in the phase II and III clinical studies of asenapine, and studies conducted in specific ethnic populations are still needed. Therefore, the study described herein was conducted to confirm the efficacy and safety of asenapine in Asian patients experiencing an acute exacerbation of schizophrenia.

## Methods

### Patients

The study was conducted from May 2010 to April 2014 at 112 centers: Japan, 81 sites; Taiwan, 15 sites; and Korea, 16 sites. Male and female patients aged 20–64 years with a *Diagnostic and Statistical Manual of Mental Disorders*, Fourth Edition, Text Revision (DSM-IV-TR) (American Psychiatric Association 2000) diagnosis of schizophrenia of paranoid (295.30), disorganized (295.10), catatonic (295.20), or undifferentiated (295.90) subtypes were eligible for randomization. The current acute exacerbation of schizophrenia had to be of ≤2 months duration, with symptoms that represented a dramatic and substantial change from the state prior to the exacerbation, and there was a requirement for a change in medication or dosage to treat new or worsened positive symptoms. Other key inclusion criteria were a PANSS total score ≥60, with scores of ≥4 in two or more of five items on the PANSS positive subscale (delusions, conceptual disorganization, hallucinatory behavior, grandiosity, suspiciousness/persecution) at the initial screening assessment and at baseline, and a score of ≥4 on the clinical global impressions-severity of illness (CGI-S) scale (Guy 1976) at baseline. Patients who had received previous anti-psychotic medication for a prior episode of acute exacerbation of schizophrenia were required to have had a positive response. The use of all prohibited concomitant medications (anti-psychotics, anti-depressants, mood stabilizers, anti-epileptics, monoamine oxidase inhibitors, St. John’s Wort, anti-emetics that are dopamine antagonists, and traditional herbal medication) had to be discontinued, with the last dose taken no later than the evening prior to the baseline visit, for inclusion in the trial (for depot neuroleptic, discontinuation must have occurred more than 3 months prior to randomization).

Patients with a diagnosis of schizoaffective disorder (295.70), schizophrenia of residual subtype (295.60), schizophreniform disorder (295.40), or a psychiatric disorder other than schizophrenia were excluded from the study. Women who were pregnant were ineligible for inclusion in the trial. Patients were also excluded if they had taken any experimental medication within 12 weeks before baseline, were defined as having treatment-refractory schizophrenia, had received treatment with ≥3 anti-psychotic drugs within the previous month, or had an uncontrolled, unstable clinically significant medical condition (e.g., renal, endocrine, hepatic, respiratory, cardiovascular, hematologic, immunologic or cerebrovascular disease, or malignancy) or abnormal laboratory, vital sign, physical examination, or electrocardiogram (ECG) findings at screening. Other exclusion criteria included the following: current (past 6 months) substance abuse; a body mass index (BMI) <16.0 or >35.0 at baseline; a diagnosis of Parkinson’s disease; a history of or current treatment for narrow angle glaucoma; a history of any seizure disorder beyond childhood; a history of allergy or sensitivity to drugs such as psychotropics and anti-psychotics; a ≥20 % decrease in PANSS total score from screening to baseline; imminent risk of self-harm or harm to others; current involuntary inpatient confinement; known diagnosis of borderline personality disorder, mental retardation, or organic brain disorder; and previous treatment with asenapine.

Patients classed as treatment-refractory (has been treated with at least two different atypical anti-psychotic agents at dosages equivalent to or greater than 600 mg/day of chlorpromazine [12 mg/day of haloperidol] for more than 4 weeks, each without clinical response, or has received clozapine for 12 weeks immediately preceding the screening) and those who received treatment with three or more anti-psychotic drugs, or dose equivalents higher than 18 mg/day of haloperidol (equivalent 900 mg/day of chlorpromazine) within 1 month prior to randomization were also excluded.

A number of measures were implemented to minimize the risks to patients randomized to placebo: hospitalization for a minimum 3 weeks, with the possibility of extending the hospitalization to 6 weeks if the patient was not clinically stable enough to be discharged; regular assessment of symptom severity; requirement that a caregiver oversees the patient’s compliance with trial medication during outpatient dosing; allowance of concomitant benzodiazepines to treat agitation and anxiety; and exclusion of high-risk subjects including those that are actively suicidal, homicidal, under involuntary commitment, or have a recent history of aggressive behavior.

### Randomization and treatment

The study consisted of a 3- to 7-day washout/screening period, a 6-week double-blind treatment phase, and a follow-up period. Placebo was administered single blind for the duration of the screening period, and patients were tapered off their pre-study anti-psychotic medication, anti-depressant medication, and any anti-parkinsonian drugs to treat extrapyramidal symptoms (EPS).

After the baseline assessments were completed, patients considered eligible by an investigator were randomized (1:1:1) to receive sublingual asenapine 5 mg bid, 10 mg bid, or placebo. The allocation of patients was performed using permuted-block randomization (block size of 6 and allocation ratio of 1:1:1) using SAS. Participants and investigators remained blinded to treatment assignment, in accordance with the double-blind study design. The asenapine and placebo tablets were identical in appearance and characteristics (i.e., all tablets were fast dissolving) and had identical packaging. During the treatment period, participants received their allocated dosage of asenapine or placebo twice a day every day for 6 weeks. Tablets were administered sublingually without water.

Participants were hospitalized during screening and for the first 3 weeks of the treatment period, with the possibility of extending hospitalization to 6 weeks in the absence of a responsible caregiver to provide support and ensure compliance with study medication. Compliance during inpatient treatment was recorded by hospital staff and monitored by returned tablet counts during outpatient treatment.

Concomitant lorazepam and short-acting benzodiazepines were permitted to treat agitation and anxiety, and concomitant EPS medication was permitted if EPS worsened or appeared.

An interim analysis was built into the protocol to assess whether the trial should be discontinued (in case of lack of efficacy). In May 2012, an independent data-monitoring committee was convened to review the efficacy data for half of the planned number of participants collected up to the day of database lock (April 26, 2012). Upon review of the data, the committee concluded that continuation of the trial was appropriate.

### Efficacy measurement

The primary efficacy outcome was change in the PANSS total score from baseline to day 42 (end of treatment). Secondary efficacy outcomes included changes from baseline to end of treatment in PANSS subscale scores (positive symptoms, negative symptoms and general psychopathology), PANSS Marder factor scores (positive symptoms, negative symptoms, disorganized thought, hostility/excitement, anxiety/depression), and CGI-S scores. Additional secondary variables were the percentage of PANSS responders (those with ≥30 % decrease in the total PANSS total score from baseline to end of treatment) and the percentage of CGI-global improvement (CGI-I) responders (those who are assessed as very much improved, much improved or minimally improved by the investigator) at day 42. Post-baseline efficacy assessments were obtained on day 4 and 7 and weekly thereafter (day 14, 21, 28, 35, and 42). Efficacy assessments were performed by an investigator or a trained rater assigned by the investigator. Using the same rater for assessment of a subject throughout their participation in the study was strongly encouraged.

### Safety assessments

Key safety parameters were weight gain, body mass index (BMI), EPS, glycosylated hemoglobin (HbA1c), fasting glucose, insulin, and prolactin. For prolactin, only clinically relevant abnormal values were reported. Other safety variables were the frequency of the onset of treatment-emergent adverse events (TEAEs) and clinically relevant changes in physical findings, vital signs, electrocardiogram parameters, laboratory values, and use of anti-parkinsonian drugs.

Laboratory, ECG, and BMI assessments were made at baseline and on days 14, 28, and 42. Assessments of vital signs were conducted at baseline and on days 14, 21, 28, and 42. Physical examinations were conducted at screening and at end of treatment (day 42). The incidence, nature, and severity of AEs were recorded throughout the screening and treatment periods until 7 days after the last dose of study drug. EPS were reported as AEs and rated according to the drug-induced extrapyramidal symptoms scale (DIEPSS) (Inada 2009) at baseline and on days 21 and 42.

### Sample size calculations

Based on the results of previous phase II and III clinical trials of asenapine in patients with schizophrenia (Minassian and Young 2010; Potkin et al. 2007), the range of the difference in the change in PANSS total score from baseline to end of treatment at 42 days between the asenapine 5 mg bid and placebo groups was between −3.4 and −9.7. Referring to the study by Kane et al. (2010), we assumed that the change from baseline in PANSS total score would be −6, and the standard deviation (SD) of the total of change in PANSS score would be 20 (effect size = 0.3). The sample size required to show superiority of asenapine over placebo was estimated be 176 per group, with a two sided significance level of 5 and 80 % power. Therefore, approximately 530 participants were expected to be randomized.

### Statistical analysis

Several sets were defined for the analysis of outcomes. The subjects as treated (i.e., safety analysis set; All Subjects Treated, AST) consisted of all participants enrolled in the study who received at least part of one dose of the study drug, while the full analysis set (FAS) consisted of all participants in the AST who had a baseline and at least one post-baseline PANSS measurement.

The primary efficacy outcome was analyzed in the FAS using analysis of covariance (ANCOVA) with dropout or missing data imputed by using the last observation carried forward (LOCF). No patient had ≥6 missing PANSS items; for 5 or fewer missing PANSS items, the PANSS score was calculated by multiplying the total for the non-missing items by the total number of items and then dividing by the number of non-missing items.

The model included change from baseline in PANSS total score as a response variable, baseline PANSS total score as a covariate, and treatment groups and regions (Japan, Taiwan, and Korea) as explanatory variables. Pairwise comparisons for the primary efficacy outcome were performed in the following order: (1) asenapine 5 mg bid vs. placebo and (2) asenapine 10 mg bid vs. placebo. The result of the second comparison was considered only when the result of asenapine 5 mg bid vs. placebo was statistically significant. Further analysis using a mixed model for repeated measures (MMRM) was performed using change from baseline in PANSS total score at each visit from 7 days as the response variables and treatment groups, regions, visits, and treatment by visit interaction as the fixed effect variables and baseline PANSS total score as the covariates. Changes from baseline to end of treatment in selected secondary efficacy outcomes (PANSS subscale, Marder factor, and CGI-S scores) were also assessed using ANCOVA with LOCF. The MMRM method was also performed for PANSS subscale and Marder factor scores. PANSS and CGI-I responder rates in the placebo versus each asenapine group were compared using the Cochran-Mantel-Haenszel (CMH) test with an adjustment for region. Regarding the analysis of secondary endpoints, these were exploratory in nature and no adjustment for multiplicity was made. Safety variables in the AST were summarized using descriptive statistics. TEAEs and categorical variables for demographics were compared the placebo and each asenapine group using Fisher’s exact test. Continuous variables for demographics were compared the placebo and each asenapine group using ANOVA. A *p* value less than 0.05 was considered statistically significant (two-tailed).

## Results

**Fig. 1:**
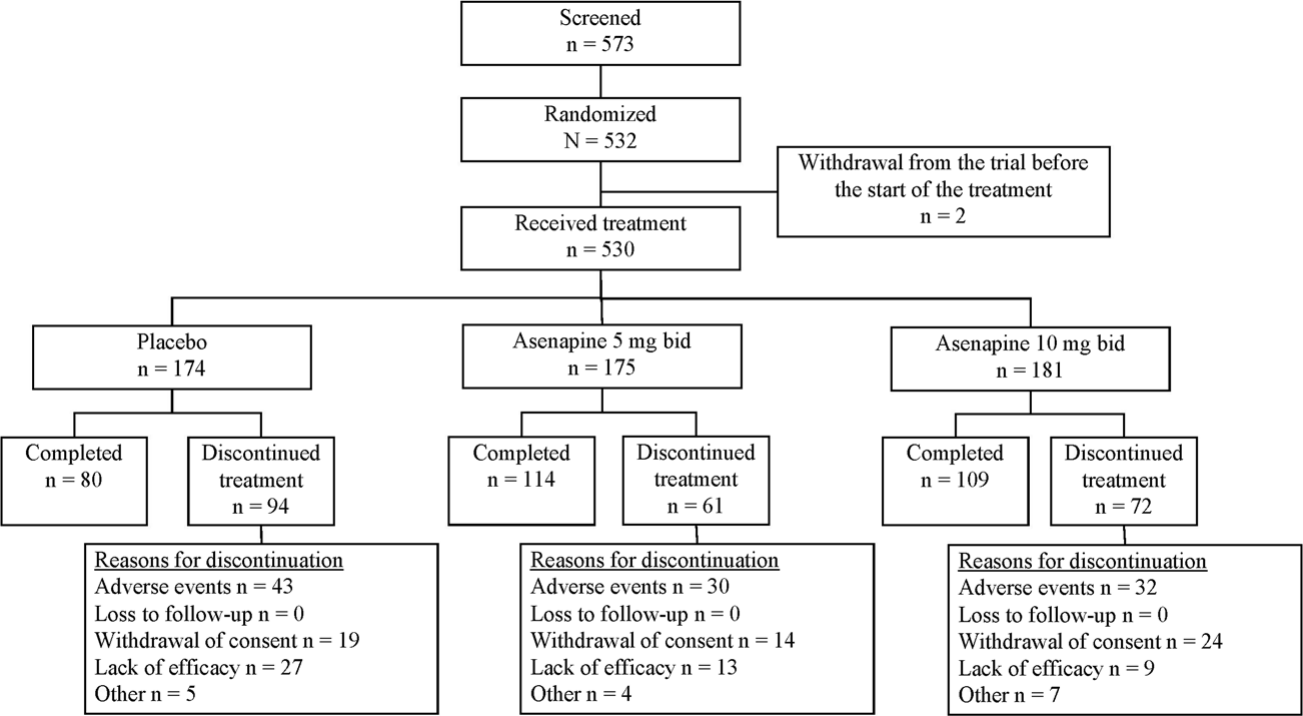
Patient disposition: numbers of patients who were screened, randomized to treatment, received treatment, and completed treatment, with reasons for discontinuation shown

Of a total of 573 patients screened, 532 participants were randomized to treatment. The AST population included 530 participants (asenapine 5 mg bid: *n* = 175; asenapine 10 mg bid: *n* = 181; placebo: *n* = 174) and the FAS included 525 participants (asenapine 5 mg bid: *n* = 173; asenapine 10 mg bid: *n* = 178; placebo: *n* = 174). The flow of participants throughout the study is presented in Fig. 1. A total of 303 participants completed treatment, with a greater percentage of those in the asenapine 5 mg bid (65 %) or 10 mg bid (60 %) groups completing the trial compared with those receiving placebo (46 %). The incidence of withdrawal due to lack of efficacy was lower for participants receiving asenapine 5 mg bid (7.4 % of treated participants) or 10 mg bid (5.0 %) than for those receiving placebo (15.5 %).

The key baseline characteristics of the AST population are presented in Table 1. No meaningful differences in baseline demographics or clinical characteristics including PANSS total scores were noted between the treatment groups. Of the concomitantly used medications, all the groups (71.3 % in the placebo, 62.9 % in the asenapine 5 mg bid, and 70.2 % in the asenapine 10 mg bid.

**Table 1:** Baseline characteristics and demographics (all patients as treated population)

Characteristic	Placebo (*n* = 174)	Asenapine 5 mg bid (*n* = 175)	Asenapine 10 mg bid (*n* = 181)	Total (*n* = 530)	*p* Value
Gender, *n* (%)					
Male	81 (46.6)	75 (42.9)	99 (54.7)	255 (48.1)	0.0722^a^
Female	93 (53.4)	100 (57.1)	82 (45.3)	275 (51.9)	
Age, years	41.11 ± 12.27	41.41 ± 11.00	41.72 ± 11.10	41.42 ± 11.45	0.8844^b^
Bodyweight, kg	62.58 ± 12.62	62.51 ± 14.15	64.28 ± 13.18	63.14 ± 13.33	0.3670^b^
BMI, kg/m^2^	23.49 ± 3.88	23.64 ± 4.05	24.15 ± 4.42	23.76 ± 4.13	0.2895^b^
Region, *n* (%)					
Japan	91 (52.3)	96 (54.9)	87 (48.1)	274 (51.7)	0.7846^a^
Taiwan	50 (28.7)	49 (28.0)	57 (31.5)	156 (29.4)	
Korea	33 (19.0)	30 (17.1)	37 (20.4)	100 (18.9)	
Schizophrenia (DSM-IV-TR) diagnosis, *n* (%)					
Paranoid	137 (78.7)	139 (79.4)	138 (76.2)	414 (78.1)	0.9850^a^
Disorganized	13 (7.5)	14 (8.0)	14 (7.7)	41 (7.7)	
Catatonic	4 (2.3)	3 (1.7)	4 (2.2)	11 (2.1)	
Undifferentiated	20 (11.5)	19 (10.9)	25 (13.8)	64 (12.1)	
Duration of current episode, *n* (%)					
<2 weeks	47 (27.0)	34 (19.4)	32 (17.7)	113 (21.3)	0.1969^a^
≥2 weeks and <1 month	47 (27.0)	59 (33.7)	55 (30.4)	161 (30.4)	
≥1 month and <2 months	80 (46.0)	81 (46.3)	94 (51.9)	255 (48.1)	
≥2 months	0	1 (0.6)	0	1 (0.2)	
PANSS total score	94.51 ± 17.26	94.15 ± 17.97	92.74 ± 17.34	93.79 ± 17.51	0.6049^b^
Concomitant medication^c^, *n* (%)					
Present	172 (98.9)	171 (97.7)	179 (98.9)		
A02 drugs for acid related disorders					
Magnesium oxide	24 (13.8)	16 (9.1)	27 (14.9)		
A06 drugs for constipation					
Sennoside a + b calcium	23 (13.2)	27 (15.4)	33 (18.2)		
Sodium picosulfate	26 (14.9)	13 (7.4)	18 (9.9)		
N02 analgesics					
Paracetamol	24 (13.8)	20 (11.4)	21 (11.6)		
N03 anti-epileptics					
Clonazepam	20 (11.5)	10 (5.7)	7 (3.9)		
Lorazepam	124 (71.3)	110 (62.9)	127 (70.2)		
N05 psycholeptics					
Olanzapine	26 (14.9)	21 (12.0)	19 (10.5)		
Aripiprazole	18 (10.3)	18 (10.3)	10 (5.5)		
Risperidone	28 (16.1)	25 (14.3)	15 (8.3)		
Etizolam	22 (12.6)	27 (15.4)	21 (11.6)		
Brotizolam	51 (29.3)	51 (29.1)	53 (29.3)		
Flunitrazepam	18 (10.3)	13 (7.4)	15 (8.3)		
Zolpidem	31 (17.8)	29 (16.6)	44 (24.3)		
Zopiclone	28 (16.1)	28 (16.0)	33 (18.2)		
N06 anti-depressants					
Escitalopram	0 (0.0)	1 (0.6)	0 (0.0)		
Escitalopram oxalate	0 (0.0)	1 (0.6)	0 (0.0)		
Fluoxetine hydrochloride	1 (0.6)	0 (0.0)	0 (0.0)		
Fluvoxamine maleate	0 (0.0)	0 (0.0)	1 (0.6)		
Sertraline hydrochloride	0 (0.0)	0 (0.0)	1 (0.6)		
Bupropion	1 (0.6)	0 (0.0))	1 (0.6)		
Mirtazapine	2 (1.1)	0 (0.0)	0 (0.0)		
Setiptiline maleate	0 (0.0)	1 (0.6)	0 (0.0)		
Trazodone	2 (1.1)	0 (0.0)	2 (1.1)		
Trazodone hydrochloride	1 (0.6)	0 (0.0)	1 (0.6)		

All data are mean ± SD unless otherwise stated

*BMI* body mass index, *DSM-IV* Diagnostic and Statistical Manual of Mental Disorders, Fourth Edition, Text Revision, *PANSS* positive and negative syndrome scale

^a^Fisher’s exact test

^b^ANOVA

^c^Drugs administered from the start of the double-blind treatment period to 7 days after the end of the study treatment (coded by WHO Drug Dictionary)

### Primary efficacy outcome: PANSS total score

**Fig. 2:**
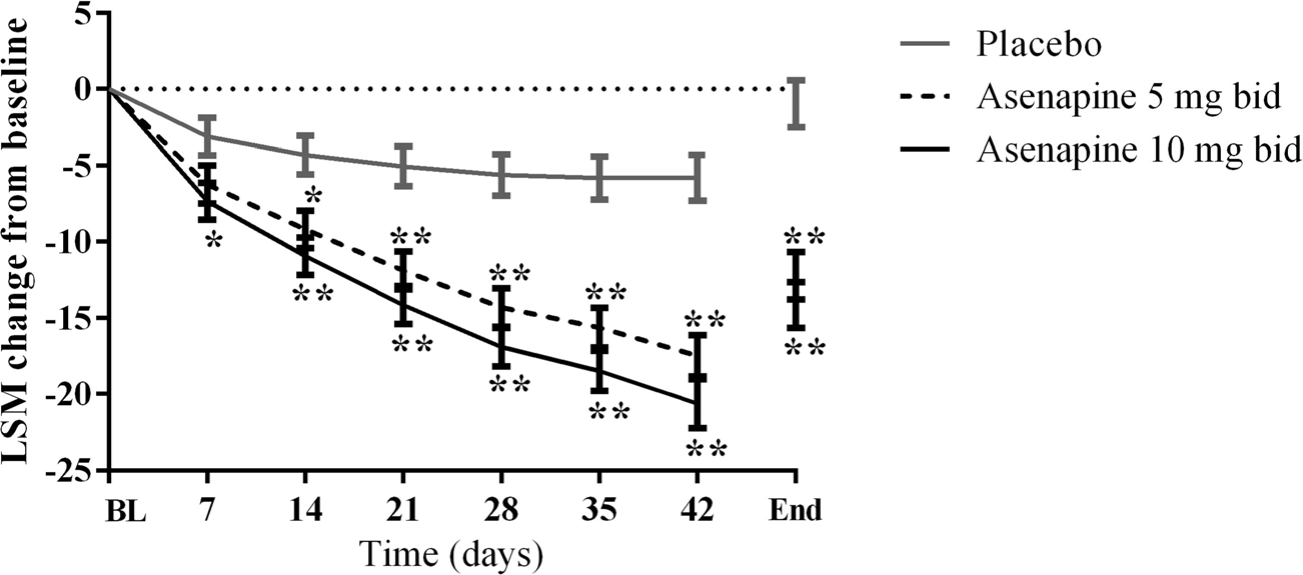
Primary efficacy outcome: change from baseline in PANSS total score over time (full analysis set population). *BL*, baseline, *LSM* least squares mean. *p < 0.05; **p < 0.01 vs. placebo

Mean PANSS total scores at baseline and at treatment end (day 42) are shown in Table 2. The least squares mean (LSM) changes from baseline in the PANSS total score at end of treatment (day 42) in the FAS were −12.24 (95 % confidence interval [CI] −15.28, −9.20), −14.17 (95 % CI −17.12, −11.22) and −0.95 (95 % CI −3.95, 2.06) in the asenapine 5 mg bid, asenapine 10 mg bid, and placebo groups, respectively. The improvements from baseline in PANSS total score were significantly greater in participants receiving asenapine 5 mg bid or asenapine 10 mg bid, compared with placebo from days 14 and 7, respectively. Overall, the efficacy profile of the asenapine 5 and 10 mg groups were similar (Table 2). Analysis of the change in PANSS total score from baseline over time using MMRM showed that improvements from baseline in PANSS total score were significantly larger in the asenapine 5 and 10 mg bid groups compared with placebo from day 14 and 7, respectively (*p* < 0.05) and were sustained through to day 42 (Fig. 2). Table 3 describes the proportion of responders using different thresholds to define response (i.e., ≥20, ≥30, ≥40, and ≥50 % decrease in PANSS total score).

**Table 2:** Positive and negative syndrome scale total scores during the study (full analysis set, last observation carried forward)

	Placebo (*n* = 174)	Asenapine 5 mg bid (*n* = 173)	Asenapine 10 mg bid (*n* = 178)	Asenapine 5 mg bid − placebo	Asenapine 10 mg bid − placebo
At baseline					
Mean	94.51	94.23	92.83		
SD	17.26	18.06	17.42		
End of treatment (day 42)					
Mean	93.38	81.84	78.60		
SD	25.30	26.10	25.01		
Change from baseline to end of treatment					
LSM	−0.95	−12.24	−14.17	−11.29	−13.22
SE	1.53	1.55	1.50	2.10	2.09
95 % CI	−3.95, 2.06	−15.28, −9.20	−17.12, −11.22	−15.42, −7.16	−17.33, −9.12
*p* value^a^	–	–	–	<0.0001	<0.0001

^a^For between-group comparisons

*bid* twice daily, *CI* confidence interval, *LSM* least squares mean, *SD* standard deviation, *SE* standard error

**Table 3:** Responder rates for change in positive and negative syndrome scale (PANSS) total scores, across different threshold values. Responder categories are separated by set limits for the magnitude of decrease in the total PANSS score from baseline to day 42/end of treatment

	Placebo (*n* = 174)	Asenapine 5 mg bid (*n* = 173)	Asenapine 10 mg bid (*n* = 178)	Asenapine 5 mg bid − placebo	Asenapine 10 mg bid − placebo
≥20 % decrease					
Responder, *n* (%)	52 (29.9)	92 (53.2)	91 (51.1)	23.3	21.2
95 % CI (%)	23.2, 37.3	45.5, 60.8	43.5, 58.7	13.2, 33.4	11.2, 31.2
≥30 % decrease					
Responder, *n* (%)	36 (20.7)	68 (39.3)	78 (43.8)	18.6	23.1
95 % CI (%)	14.9, 27.5	32.0, 47.0	36.4, 51.4	9.2, 28.1	13.7, 32.6
≥40 % decrease					
Responder, *n* (%)	21 (12.1)	41 (23.7)	56 (31.5)	11.6	19.4
95 % CI (%)	7.6, 17.9	17.6, 30.7	24.7, 38.8	3.7, 19.6	11.0, 27.8
≥50 % decrease					
Responder, *n* (%)	8 (4.6)	25 (14.5)	40 (22.5)	9.9	17.9
95 % CI (%)	2.0, 8.9	9.6, 20.6	16.6, 29.3	3.8, 15.9	11.0, 24.8

*bid* twice daily, *CI* confidence interval

### Secondary efficacy outcomes

#### Positive and negative syndrome scale subscale scores and responders

**Fig. 3:**
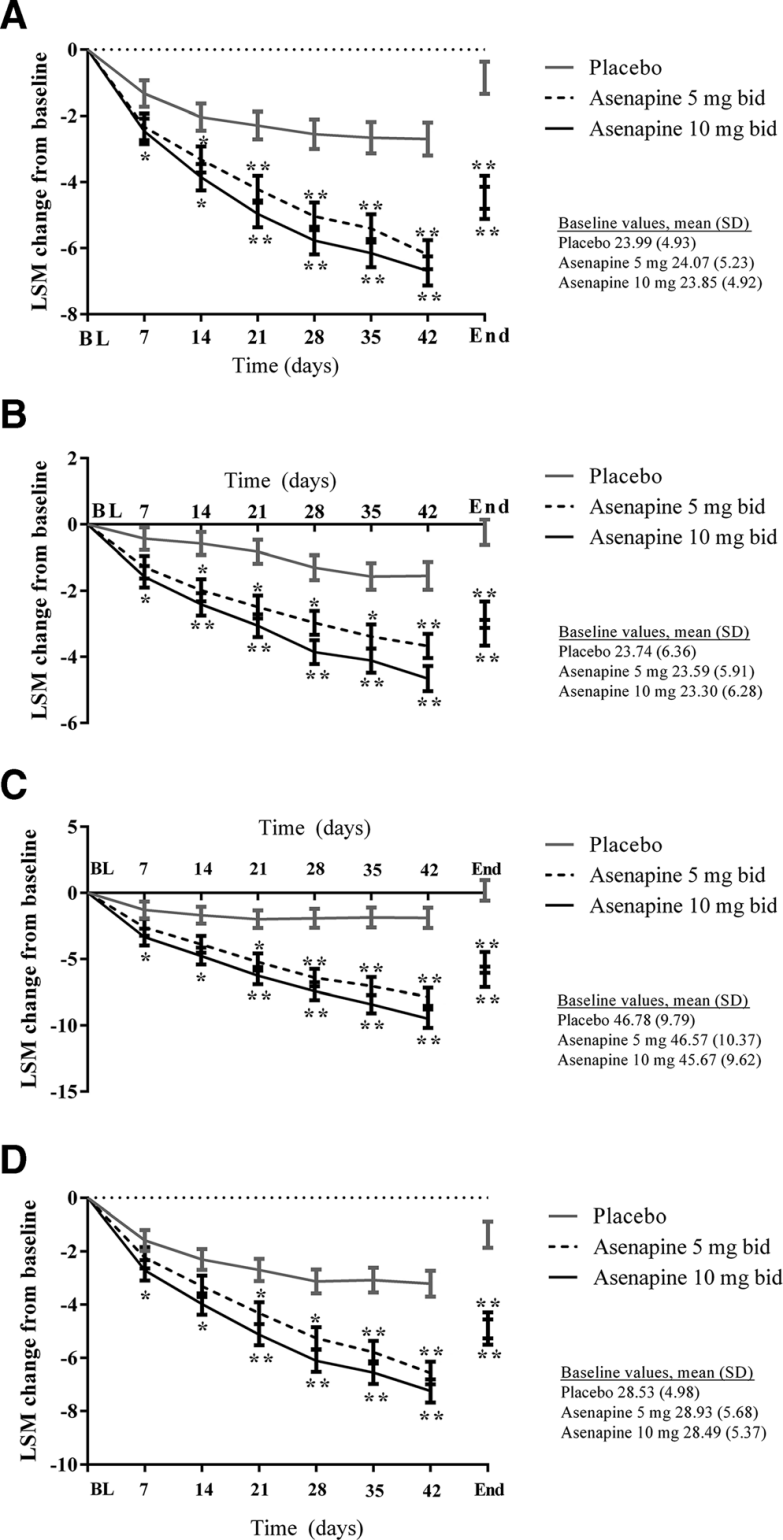

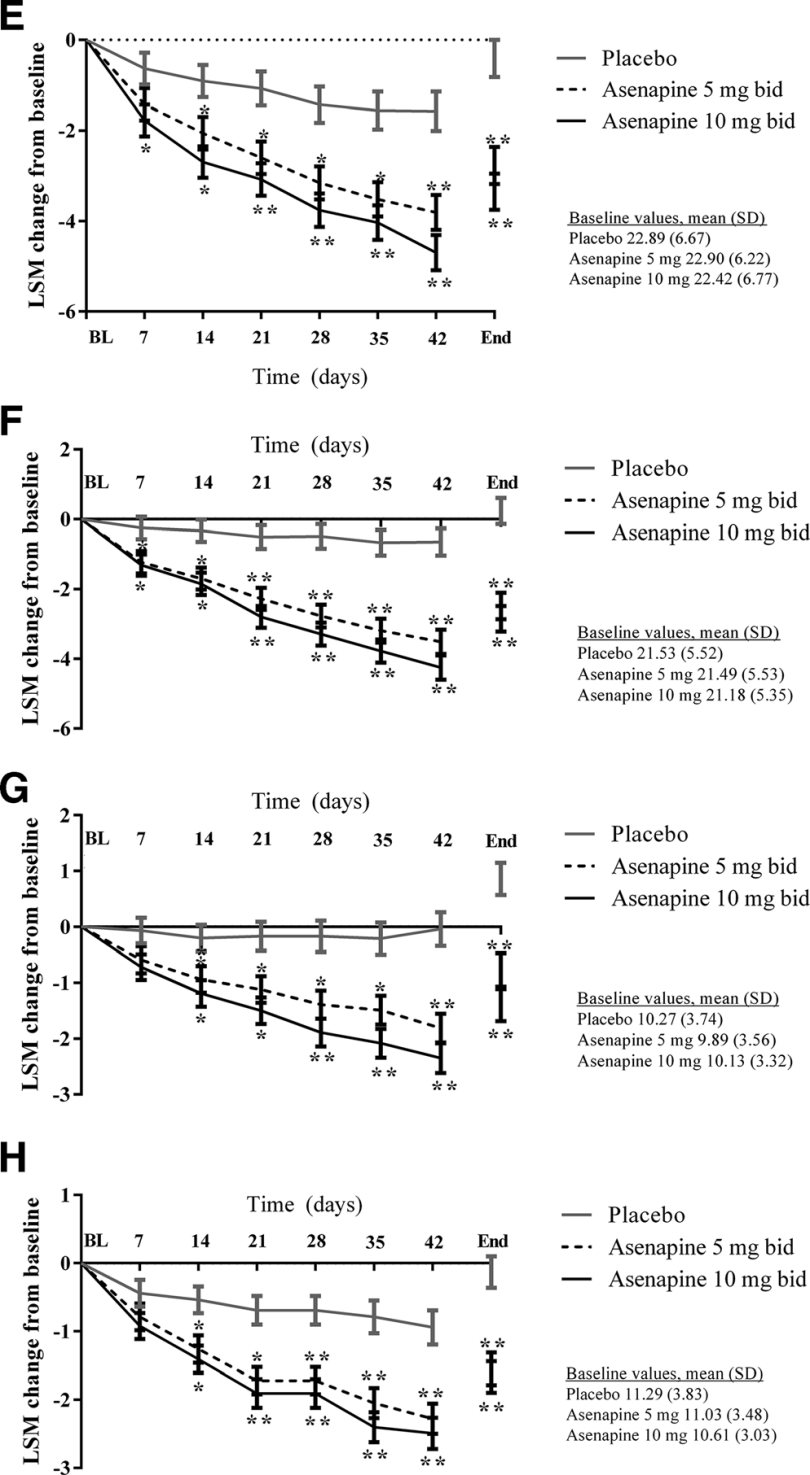
Secondary efficacy outcomes: changes from baseline in **a** PANSS positive symptom factor, **b** PANSS negative symptom factor, **c** PANSS general psychopathology score, **d** PANSS Marder positive symptom factor, **e** PANSS Marder negative symptom factor, **f** PANSS Marder disorganized thought factor, **g** PANSS Marder Hostility/Excitement Factor, and **h** PANSS Marder anxiety/depression factor. *BL* baseline, *End* end of treatment, *LSM* least squares mean, *SD* standard deviation. *p < 0.05; **p < 0.01 vs. placebo

Changes in the PANSS subscale scores and PANSS Marder factor scores supported the results of the primary efficacy outcome analysis (Fig. 3a–h), whereby significantly more participants were classified as PANSS responders (≥30 % decrease in score) at the end of treatment in the asenapine 5 mg bid (*p* = 0.0001) and 10 mg bid (*p* < 0.0001) groups compared with the placebo group. This was evident for PANSS positive, negative, and general scores and all PANSS Marder subscores.

#### Clinical global impressions-severity of illness scores and responders

CGI-S scores also supported the results of the primary efficacy outcome analysis. The percentage of CGI-I responders at end of treatment was significantly higher in both asenapine treatment groups than in the placebo group (both *p* < 0.0001).

### Safety and tolerability

There were no differences in the overall incidence of TEAEs across the treatment groups; TEAEs were reported in 81.6 % of participants receiving placebo, 84.6 % of participants receiving asenapine 5 mg bid, and 80.7 % of participants receiving asenapine 10 mg bid. Serious adverse events (SAEs) were reported in 5.7 and 2.8 % of participants in the asenapine 5 mg bid and 10 mg bid groups, and in 7.5 % of those receiving placebo. No SAE occurred in more than one subject, apart from aggravated schizophrenia, which occurred in 4.6 and 2.2 % of participants receiving asenapine 5 mg bid and 10 mg bid, respectively, and 4.6 % of the placebo group. As per withdrawals for lack of efficacy, the incidence of withdrawal due to adverse events (AEs) was lower for those receiving asenapine 5 mg bid (17.1 % of treated participants) or 10 mg bid (17.7 %), compared with those receiving placebo (24.7 %).

#### Disease exacerbation and extrapyramidal symptoms

TEAEs occurring in ≥5 % of participants in any treatment group are shown in Table 4. The most commonly reported TEAEs for asenapine were aggravated schizophrenia, akathisia, oral hypoesthesia, and somnolence. As shown in Table 4, aggravated schizophrenia occurred in at least 10 % of subjects in each treatment group but was less frequent in the asenapine groups than in the placebo group. In contrast, rates of EPS and akathisia were higher in participants receiving asenapine 5 mg (5.1 and 11.4 %) and 10 mg (7.7 and 10.5 %) bid than in those receiving placebo (1.7 and 5.2 %). The incidence rates of dizziness and increase in blood creatine phosphokinase were higher in the asenapine 10 group, but there were no differences in the other TEAEs between the treatment groups (Table 4).

**Table 4:** Incidence of treatment-emergent adverse events occurring in ≥5 % of patients in at least one treatment group (all patients as treated population)

Adverse event, n (%)	Placebo (*n* = 174)	Asenapine 5 mg bid (*n* = 175)	Asenapine 10 mg bid (*n* = 181)	*p* Value^a^
Any adverse event	142 (81.6)	148 (84.6)	146 (80.7)	0.4018
Aggravated schizophrenia	49 (28.2)	23 (13.1)	28 (15.5)	0.5488
Hypoaesthesia oral	6 (3.4)	19 (10.9)	17 (9.4)	0.7261
Akathisia	9 (5.2)	20 (11.4)	19 (10.5)	0.8657
Extrapyramidal symptoms	3 (1.7)	9 (5.1)	14 (7.7)	0.3906
Somnolence	3 (1.7)	17 (9.7)	22 (12.2)	0.5004
Headache	11 (6.3)	11 (6.3)	10 (5.5)	0.8243
Constipation	11 (6.3)	10 (5.7)	13 (7.2)	0.6683
Dizziness	5 (2.9)	7 (4.0)	17 (9.4)	0.0560
Sedation	2 (1.1)	4 (2.3)	9 (5.0)	0.2588
Vomiting	6 (3.4)	8 (4.6)	9 (5.0)	1.0000
Blood creatine phosphokinase increased	4 (2.3)	3 (1.7)	12 (6.6)	0.0318
Insomnia	21 (12.1)	17 (9.7)	14 (7.7)	0.5748
Nasopharyngitis	8 (4.6)	13 (7.4)	11 (6.1)	0.6755

Comparison between the 5 and 10 mg groups

^a^Fisher’s exact test

#### Weight

There was a mean (± standard deviation [SD]) change in weight of −1.76 ± 2.45 kg for placebo, +0.42 ± 2.65 kg for asenapine 5 mg bid, and +0.81 ± 2.89 kg for asenapine 10 mg bid. Clinically significant weight gain (≥7 % of baseline body weight) was reported in 4.7 to 7.3 % of participants in the asenapine groups and none in the placebo group. BMI decreased by 0.66 ± 0.91 kg/m^2^ with placebo and increased by 0.16 ± 1.03 and 0.32 ± 1.09 kg/m^2^ with asenapine 5 mg bid and 10 mg bid.

#### Laboratory values and vital signs

There were no clinically significant differences in laboratory values and vital signs between the treatment groups. Mean ± SD changes from baseline in insulin were 2.37 ± 14.08, 2.24 ± 13.78, and 0.22 ± 20.34 μIU/mL with asenapine 5 mg bid and 10 mg bid and placebo, respectively; mean ± SD changes in fasting glucose were −0.03 ± 0.85, 0.11 ± 0.77, and 0.18 ± 0.97 mmol/L; and mean ± SD changes in HbA1c were −0.01 % ± 0.41 %, −0.01 ± 0.49 % and −0.04 ± 0.45 %.

Mean ± SD changes from baseline in prolactin were −17.92 ± 45.31, −13.27 ± 43.93 and −27.79 ± 46.25 μg/L with asenapine 5 mg bid and 10 mg bid and placebo, respectively. The percentage of participants who had normal prolactin levels at baseline but levels above the reference range at the end of treatment (day 42) was higher in those receiving asenapine 5 mg bid (27.9 %) and asenapine 10 mg bid (31.7 %) than in those receiving placebo (10.3 %).

No adverse event related to QT prolongation (SMQ) was observed during the monitoring of the current trial; however, the data are not shown.

### Stratified analyses

Given that the study was performed over a number of different sites and countries, the potential for a country-by-treatment interaction was examined, but no significant influences were found (i.e., results were *p* > 0.05). Furthermore, stratified analyses were performed for sex, disease duration, and schizophrenia subtypes; the results of each indicated that there were no demographic or disease subtype factors that influenced the overall results.

## Discussion

In this randomized, double-blind clinical trial, asenapine 5 and 10 mg bid was significantly more effective than placebo in treating acute schizophrenia in Asian patients, as demonstrated by statistically significant improvements from baseline in PANSS total scores. In addition, asenapine 5 and 10 mg bid was effective in controlling both positive and negative symptoms, providing statistically significant improvements from baseline in PANSS positive and negative scores compared with placebo (by 11–13 points), as well as significant improvements in general scores and PANSS Marder 5 factor scores. Moreover, the asenapine treatment groups showed significant improvements versus placebo in other secondary efficacy variables (though it should be noted that these secondary variables were only part of an exploratory analysis); overall the efficacy profiles of the asenapine 5 and 10 mg groups appeared to be similar. Asenapine was safe and well tolerated, with minimal impact on body weight and no notable effects on other metabolic parameters. The incidence of discontinuation because of AEs or lack of efficacy was greater in the placebo group than in the asenapine treatment groups. With the exception of elevated blood creatine phosphokinase and increased dizziness, there were no differences between the 5 and 10 mg groups for TEAEs.

Results of the current study, conducted in Asian countries, complement the robustly positive results of two similarly designed 6-week studies conducted in North America and Europe in patients with an acute exacerbation of schizophrenia (Kane et al. 2010; Potkin et al. 2007). While the study by Kane et al. found significantly greater reductions in mean PANSS total scores, no significant difference from placebo was observed in negative scores, or hostility/excitement subscores (Kane et al. 2010). However, in our study, asenapine significantly reduced the scores on the negative factor and hostility/excitement factor, suggesting that asenapine has efficacy across a broad range of symptoms in schizophrenia. Two other short-term trials have been conducted with asenapine in the 5–10 mg bid dose range: one was negative (active control, but not asenapine, separated from placebo) and one failed (neither asenapine nor active control separated from placebo) (Citrome 2014b; Szegedi et al. 2012). A meta-analysis of pooled data from all four trials found asenapine to be superior to placebo with regard to mean change in PANSS total score (Szegedi et al. 2012). Both asenapine doses were safe and well tolerated, with minimal effects on weight and no notable effects on metabolic parameters, in all studies.

In one of the previous asenapine studies, a high placebo response rate was reported; the authors suggested that this may have contributed to the finding that asenapine 10 mg bid did not have an advantage over placebo in that trial (Kane et al. 2010). Low effect sizes versus placebo are also illustrated in the previously mentioned failed and negative studies, reflecting the finding that placebo response tended to be larger than historically expected (Szegedi et al. 2012). High placebo response rates underscore the need to demonstrate that active treatment can separate from placebo (Kane et al. 2010). In order to exclude initial placebo responders in the present study, oral placebo tablets were administered during the screening period. Restriction of the duration of the current acute exacerbation of schizophrenia to ≤2 months may have contributed to the low placebo response rate and efficacy separation of the asenapine treatment groups from placebo seen in this trial. In the present trial, asenapine 5 mg bid and 10 mg bid were both found to be significantly effective in reducing PANSS scores compared with placebo, which is clearly in line with the previous pooled analysis of placebo-controlled trials of asenapine in acute schizophrenia showing that the two doses had similar efficacy (Friberg et al. 2009).

The results of the present study demonstrate that asenapine is well tolerated, with rates of SAEs and withdrawals due to AEs lower in both active treatment groups compared with placebo. The AE profile of asenapine seen in this study was as expected; reviews of the literature show that asenapine treatment is associated with EPS and akathisia, increases in prolactin versus placebo, oral hypoesthesia, and weight gain (a known class effect) (Citrome 2014b), and the results reported herein confirm that asenapine recipients experience these AEs more frequently than those receiving placebo. However, other active controlled studies suggest that the rates of EPS and akathisia vary depending on the drug, and that asenapine induces these events at a lower rate than some anti-psychotics (Citrome 2014b). Similarly, the effect of asenapine on weight has been reported as modest compared with other anti-psychotics such as olanzapine (Citrome 2014b). In the present study, only modest weight gain was observed in the asenapine group. Asenapine’s unique functional activities at diverse neurotransmitter receptors, including various serotonin receptors, may contribute to the relatively benign effects on body weight (Tarazi and Neill 2013).

Limitations of this study include the absence of an active control arm and ethical questions regarding the use of placebo in patients with acute schizophrenia. Another limitation of this study was the reliance on pill counts to monitor adherence rather than more reliable measures, such as measurement of blood drug levels.

In conclusion, results of this double-blind, placebo-controlled 6-week study showed that asenapine 5 mg bid and 10 mg bid are effective and well tolerated in the treatment of Asian patients with an acute exacerbation of schizophrenia.
